# Non-linear association between long-term air pollution exposure and risk of metabolic dysfunction-associated steatotic liver disease

**DOI:** 10.1265/ehpm.23-00271

**Published:** 2024-02-10

**Authors:** Wei-Chun Cheng, Pei-Yi Wong, Chih-Da Wu, Pin-Nan Cheng, Pei-Chen Lee, Chung-Yi Li

**Affiliations:** 1 Department of Public Health, College of Medicine, National Cheng Kung University, Tainan, Taiwan; 2 Department of Internal Medicine, College of Medicine, National Cheng Kung University, Tainan, Taiwan; 3 Department of Gastroenterology and Hepatology, Tainan Hospital, Ministry of Health and Welfare, Tainan, Taiwan; 4 Department of Environmental and Occupational Health, National Cheng Kung University, Tainan, Taiwan; 5 Department of Geomatics, National Cheng Kung University, Tainan, Taiwan; 6 National Institute of Environmental Health Sciences, National Health Research Institutes, Miaoli, Taiwan; 7 Innovation and Development Center of Sustainable Agriculture, National Chung Hsing University, Taichung, Taiwan; 8 Department of Public Health, College of Public Health, China Medical University, Taichung, Taiwan; 9 Department of Healthcare Administration, College of Medical and Health Science, Asia University, Taichung, Taiwan

**Keywords:** Metabolic dysfunction-associated steatotic liver disease (MASLD), Air pollutants, Particular matters, Risk factors, Epidemiologic study, Restricted cubic spline (RCS)

## Abstract

**Background:**

Metabolic Dysfunction-associated Steatotic Liver Disease (MASLD) has become a global epidemic, and air pollution has been identified as a potential risk factor. This study aims to investigate the non-linear relationship between ambient air pollution and MASLD prevalence.

**Method:**

In this cross-sectional study, participants undergoing health checkups were assessed for three-year average air pollution exposure. MASLD diagnosis required hepatic steatosis with at least 1 out of 5 cardiometabolic criteria. A stepwise approach combining data visualization and regression modeling was used to determine the most appropriate link function between each of the six air pollutants and MASLD. A covariate-adjusted six-pollutant model was constructed accordingly.

**Results:**

A total of 131,592 participants were included, with 40.6% met the criteria of MASLD. “Threshold link function,” “interaction link function,” and “restricted cubic spline (RCS) link functions” best-fitted associations between MASLD and PM_2.5_, PM_10_/CO, and O_3_ /SO_2_/NO_2_, respectively. In the six-pollutant model, significant positive associations were observed when pollutant concentrations were over: 34.64 µg/m^3^ for PM_2.5_, 57.93 µg/m^3^ for PM_10_, 56 µg/m^3^ for O_3_, below 643.6 µg/m^3^ for CO, and within 33 and 48 µg/m^3^ for NO_2_. The six-pollutant model using these best-fitted link functions demonstrated superior model fitting compared to exposure-categorized model or linear link function model assuming proportionality of odds.

**Conclusion:**

Non-linear associations were found between air pollutants and MASLD prevalence. PM_2.5_, PM_10_, O_3_, CO, and NO_2_ exhibited positive associations with MASLD in specific concentration ranges, highlighting the need to consider non-linear relationships in assessing the impact of air pollution on MASLD.

**Supplementary information:**

The online version contains supplementary material available at https://doi.org/10.1265/ehpm.23-00271.

## Graphical Abstract

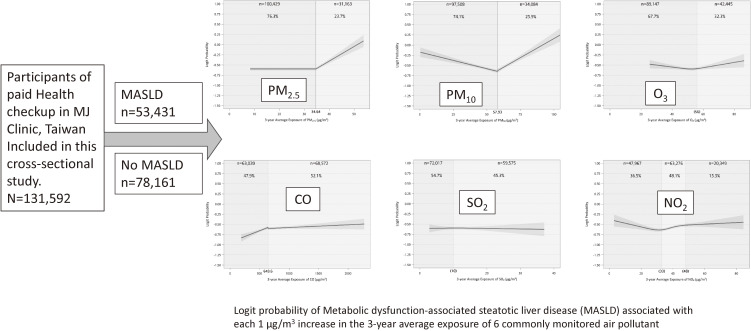

## Introduction

Non-alcoholic fatty liver disease (NAFLD) is a complex systemic inflammatory and metabolic disease majorly defined by its liver manifestation [1, 2]. The updated definition, metabolic dysfunction-associated fatty liver disease (MAFLD), was established to emphasize on the pathophysiological nature of metabolic dysfunctions [3] and related adverse clinical outcomes [4, 5]. The prevalence of fatty liver/steatotic liver disease has been growing rapidly over time [6–8], making it a major contributing factors to the loss of life-years worldwide [9]. Thus in 2023, the nomenclature and the diagnostic criteria were updated by three large pan-national liver associations. The newly proposed metabolic dysfunction-associated steatotic liver disease (MASLD) had much clear definitions for epidemiological studies [10].

In addition to lifestyle and behavior risk factors such as alcohol, smoking and dietary imbalance, air pollution has recently been recognized as one of the potential risk factors for liver diseases, particularly in complex liver diseases like MASLD [11, 12]. Prior studies have indicated that nitro dioxide (NO_2_) is associated with increased oxidative stress and biomarkers of hepatic inflammation [11, 13]. Additionally, particulate matter (PM), especially those with diameter smaller than 2.5 µm (PM_2.5_), can penetrate the alveoli and trigger systemic inflammatory responses [14–16]. Long-term exposure to elevated level of PM_2.5_ have been found to be associated with insulin resistant, metabolic syndrome, and possibly MASLD [17–20]. However, two recent large-scale studies have suggested a possible non-linear relationship between air pollutant exposure and the risk of MASLD. In a landmark study by Guo and colleagues, a non-linear association was reported between long term exposure to elevated PM_2.5_ concentration and the odds ratio (OR) for MAFLD [17]. Similarly, Sun et al, used a piecewise analysis with break points to model the hazard ratio for NAFLD in relation to PM_2.5_ [18]. Furthermore, no previous reports have explored the potential risk of MASLD associated with the combined effects of commonly monitored air pollutants. The objective of this study was to investigate the existence and form of non-linear relationships between six regularly monitored ambient air pollutants, individually and collectively, and prevalence of MASLD.

## Materials and methods

### Study design and covariates

A cohort of paid health checkup participants from the MJ clinic, were included in this cross-sectional study. The study was approved by the Institutional Review Board of National Cheng Kung University Hospital, Tainan, Taiwan (Approval Number: B-ER-110-456) with a waiver of informed consent. Participants provided detailed demographic, lifestyle and medical history information through self-administered questionnaires [21]. Socio-demographic characteristics and lifestyle were obtained from self-administered questionnaires on the day of health checkup, including vegetable, fruit, sugar drink and fried food intake amount and frequency (seldom, moderate and frequent intake), and cigarette smoking status (never, former or current). As for alcohol drinking, those who consumed two or more alcoholic drinks per day on three or more days a week for more than 1 year [22] (ie, evidently took more than 70 to 140 gm alcohol intake per week) were considered as having excessive alcohol drinking. Participants with excessive alcohol consumption were excluded in current study, as hepatic steatosis in this group may be related to alcohol, they should be classified as MASLD with increased alcohol intake (MetALD) or alcohol-related liver disease (ALD) [10]. This distinction is crucial because the disease course in ALD or MetALD may differ significantly from MASLD.

Regular exercise was defined as taking at least half an hour of exercise once per 2–3 days. Anthropometric measurements and blood tests were conducted and reported by standard protocols of MJ Health Management Institution. The fasting glucose and lipid profile were taken under fasting state. Diabetes mellitus (DM) was defined as a fasting serum glucose ≥126 mg/dL, or HbA1c ≥6.5% [23] or self-reported history. Hypertension (HTN) was defined as grade 1: systolic blood pressure (BP) between 130 to 139 mmHg or diastolic BP between 80 to 89 mmHg; grade 2: ≥140/90 mmHg or under specific drug treatment [24, 25]. Dyslipidemia was defined as either one of the following: (1) total cholesterol ≥240 mg/dL; (2) high-density lipoprotein cholesterol <40 mg/dL; (3) triglyceride ≥200 mg/dL; (4) low-density lipoprotein cholesterol ≥160 mg/dL; or (5) under specific drug treatment [26–28]. Abnormal liver function test was defined as elevated serum alanine aminotransferase (ALT) above upper limit of normal range. For missing information in major socioeconomic risk factors for steatotic liver disease (marriage, education status and annual household income), they were coded as “unreported” and analyzed as a unique category. The proportion of missing information in the other variables (eg, smoking, diet, exercise…, etc) was less than 3% and was accounted for by imputing the most prevalent category.

### Study population and inclusion/exclusion criteria

Records of all participants attending health checkups from 2010 to 2017 were retrieved (N = 382,914). We excluded 17,642 records (4.6%) from the analysis due to missing information of home address, living at regions without reliable air pollution exposure data (e.g., living in remote islands), or missing sonography data to determine hepatic steatosis status. For the participants with more than one health checkup record during the study period, the earliest record (i.e., index health checkup) was retained, leaving 195,625 participants to the study cohort.

**Fig. 1:**
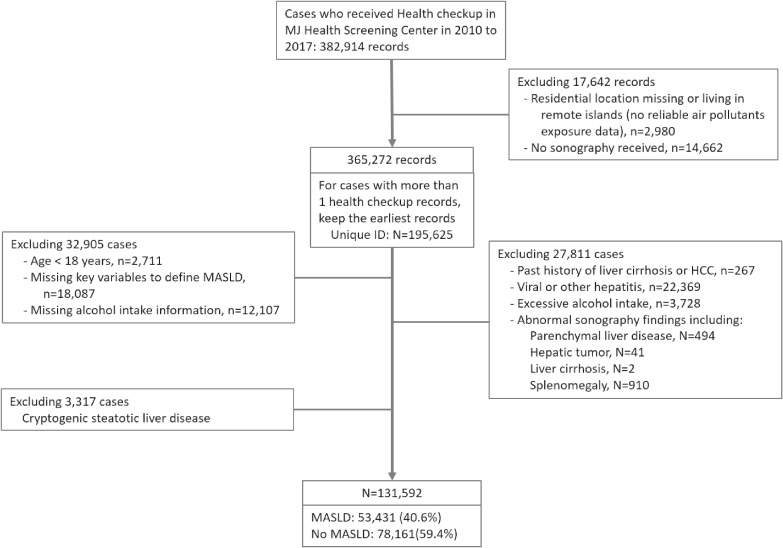
The flow diagram of enrolling study participants of this study. HCC: hepatocellular carcinoma, MASLD: metabolic dysfunction-associated steatotic liver disease

Exclusion criteria also included: age <18 years (n = 2,711), missing key information to define MASLD (n = 18,087) and missing alcohol intake information (n = 12,107). We further excluded participants with self-reported past histories of liver cirrhosis or hepatocellular carcinoma (n = 267), with viral or other hepatitis (n = 22,369), with excessive alcohol intake (n = 3,728, see “Covariates” session) and those with significant sonography abnormalities (n = 1,447.) In the remaining participants, 3,317 categorized as cryptogenic steatotic liver disease were not analyzed in our study. Finally, 131,592 participants were included in the current analysis. Among them, 53,431 (40.6%) met the definition of MASLD. The flow chart of study participants’ enrollment is illustrated in Fig. 1.

### Assessment of residential air pollution exposure

The monthly average concentration for each of the 6 major air pollutants including PM with aerodynamic diameter ≤2.5 µm (PM_2.5_), PM with aerodynamic diameter ≤10 µm (PM_10_), carbon monoxide (CO), ozone (O_3_), sulfur dioxide (SO_2_), and NO_2_ from all 74 air quality monitoring stations in Taiwan were retrieved from the air pollution database supervised by Taiwan Environment Protection Administration (TWEPA). Information on the measurement instrument, method and sensitivity for air quality monitoring was available from the TWEPA website [29]. The spatial-temporal concentration distribution of each pollutant was estimated by land-use regression in combination with XGBoost machine learning algorithm (LUR-XGBoost) or Hybrid Kirging-LUR with XGboost algorithm with a 50-m × 50-m grid resolution. The performance of these algorithm demonstrated 10-fold cross-validation r^2^ as 0.94 for PM_2.5_, 0.84 for CO, 0.90 for NO_2_ and 0.88 for O_3_ with similarly excellent performance for external validity [30–33]. Exposure levels of various air pollutants were assessed based on the geocode of each participant’s home address. We calculated 1- to 4-year average exposure levels prior to the date of index health checkup for each study participant (Table S1) and chose 3-year average of air pollutant concentration to indicate long-term air pollutant exposures in this study [17]. To account for the observed trend of gradually decreasing air pollutant concentrations over time, we included the year of each participant’s enrollment in our multivariate models for adjustment.

### Definition of metabolic dysfunction-associated steatotic liver disease (MASLD)

MASLD was defined as cases who had hepatic steatosis combined with at least one of the 5 cardiometabolic criteria [10]: 1) body mass index (BMI) ≧23 Kg/m^2^, or waist circumference (WC) >94 cm in men or WC >80 cm in women; 2) fasting serum glucose ≥100 mg/dL or HbA1c ≥5.7% or history of type 2 diabetes or under drug treatment; 3) BP ≥130/85 mmHg or under specific antihypertensive drug treatment; 4) plasma triglycerides (TG) ≥150 mg/dL or under lipid lowering treatment; 5) plasma high density lipoprotein-cholesterol (HDL) ≤40 mg/dL for men or 50 mg/dL for women or under lipid lowering treatment. Hepatic steatosis was determined on abdominal ultrasonography performed by well-trained clinicians at MJ clinics [34].

### Identification of potential non-linearity relationship of air pollution exposure and MASLD

To determine the non-linear link functions between air pollutant exposure and MASLD [17, 18], we combined data visualization with regression modeling to determine the best-fitted link function as the following:

1) We initially assessed the proportionality or linearity of air pollution exposure and MASLD odds using the Box-Tidwell test for each specific pollutant by testing for the significance of interaction between continuous air pollutant concentrations and their corresponding natural log [35].
2) If the proportional odds assumption held, a ‘linear link function’ was identified. For non-proportional relationships, we generated logit probability plots of MASLD by dividing air pollution concentrations into 100 percentile bins.
3) We used an iterative method minimizing non-linear least squares to identify inflection points and estimated the slopes of linear logistic regression models before and after these points, performed with the SAS procedure NLIN (SAS Institute, Cary, NC). Next, several non-linear link functions were explored and compared based on the Akaike Information Criterion (AIC). These functions included:
3.1) “Threshold link function”: assigned exposure below the inflection point as uniform baseline hazard (slope = 0) and a linear link function with proportional odds above the inflection point.
3.2) “Interaction link function”: two different linear link functions for exposure above and below the inflection point. An additional variable was then introduced, where a value of 0 and 1 was assigned to the participants with exposure below and above the inflection point, respectively. The original continuous exposure level, the newly created dummy variable, and their interaction term were then simultaneously included in the model.
3.3) “Restricted cubic spline (RCS) link function”: piecewise polynomials capturing curving relationships between exposure and logit probability of MASLD. A default RCS function with 4 knots was initially utilized with the location of knots determined according to previous literature [36]. If the 4-knot RCS model proved to be superior to other linear-based models, RCS functions with 3 to 7 knots were then sequentially evaluated to identify the optimal number of knots.

Any pair of the above link functions was considered equally fitted if a difference in AIC was 10 or less between link functions. In such circumstances, the principle of parsimony applied. The best-fitted link function was chosen as the following order: “linear link function”, “threshold link function”, “interaction link function” and “RCS link function”, with fewer knots was considered simpler.

To identify optimal link functions for each air pollutant and MASLD, we compared observed and predicted logit probabilities in both unadjusted and covariate-adjusted one-pollutant logistic regression models. Subsequently, the covariate-adjusted six-pollutant model was formed using these optimal link functions.

### Statistical analysis

Descriptive statistics were presented mean ± standard deviation (SD) for continuous variables and number/percentage for categorical variables when comparing characteristics of individuals with and without MASLD. One-pollutant and six-pollutant logistic regression models, with best-fitted link functions, were presented with estimated covariate adjusted odds ratios (aORs) and 95% CIs. Pollutant exposure was analyzed both continuously and discretely, categorized by inflection points from best-fit link functions. For multiple logistic regression models, hypertension, diabetes mellitus and dyslipidemia were considered as part of the outcome variable within MASLD definition and were left unadjusted. Sensitivity analyses were conducted to assess the robustness of results: 1) The 6-pollutant model with linear link function for all 6 pollutants; 2) A 5-pollutant model that excluded PM_10_, due to its relatively high correlation with PM_2.5_ and SO_2_. All analyses were performed using SAS version 9.4 (SAS Institute, Cary, NC), with a significance level of α = 0.05.

## Results

In the 131,592 health checkup participants included, 53,431 (40.6%) had MASLD (Fig. 1). Socio-demographic characteristics and clinical parameters for participants with and without MASLD were compared and shown in Table 1. Participants with MASLD were older, predominantly male, and had higher percentage of being married. Furthermore, they exhibited a higher prevalence of current or former smoking and alcohol intake. Table 1 also showed the 3-year average exposure to 6 air pollutants prior to the index checkup. Compared to those without MASLD, participants with MASLD tended to have greater exposure levels of all 6 commonly monitored air pollutants.

**Table 1:** The demographic variables and clinical parameters of people with and without MASLD.

	**No MASLD** **(n = 78,161)**	**MASLD** **(n = 53,431)**

	**Mean ± SD / n (%)**	**Mean ± SD / n (%)**
Age, years	37.9 ± 11.9	44.3 ± 12.5
Sex		
Male	27,937 (35.7)	35,259 (66.0)
Marriage		
Single	34,003 (43.5)	14,818 (27.7)
Married	40,278 (51.5)	35,823 (67.1)
Unreported	3,880 (5.0)	2,790 (5.2)
Education		
Illiterate	574 (0.7)	848 (1.6)
Elementary or middle schools	4,871 (6.2)	6,468 (12.1)
High schools	25,695 (32.9)	19,390 (36.3)
College or higher	45,795 (58.6)	25,720 (48.1)
Unreported	1,226 (1.6)	1,005 (1.9)
Annual household income		
<28,000 USD	32,825 (42.0)	20,517 (38.4)
28,000 to 70,000 USD	30,204 (38.7)	22,093 (41.3)
≧70,000 USD	5,182 (6.6)	4,460 (8.4)
Unreported	9,950 (12.7)	6,361 (11.9)
Smoking		
Never	64,087 (82.0)	37,668 (70.5)
Former smoker	3,705 (4.7)	4,716 (8.8)
Current smoker	10,369 (13.3)	11,047 (20.7)
Alcohol drinking		
Never	68,135 (87.2)	44,101 (82.5)
Former	1,402 (1.8)	1,460 (2.7)
Current	8,624 (11.0)	7,870 (14.7)
Vegetable Intake		
Seldom	13,581 (17.4)	9,087 (17.0)
Moderate	46,646 (59.7)	32,452 (60.7)
Frequent	17,934 (22.9)	11,892 (22.3)
Fruit intake		
Seldom	26,169 (33.5)	16,531 (31.0)
Moderate	42,420 (54.3)	29,901 (56.0)
Frequent	9,572 (12.3)	6,999 (13.1)
Sugary drink intake		
Seldom	27,053 (34.6)	19,414 (36.3)
Moderate	38,167 (48.8)	25,266 (47.3)
Frequent	12,941 (16.6)	8,751 (16.4)
Fried food intake		
Seldom	23,240 (29.7)	15,313 (28.7)
Moderate	41,657 (53.3)	27,961 (52.3)
Frequent	13,264 (17.0)	10,157 (19.0)
Habit of regular exercise	25,329 (32.4)	17,805 (33.3)
Body mass index (BMI), kg/m^2^	21.3 ± 2.6	26.2 ± 3.4
Waist circumference (WC), cm		
Male	77.7 ± 6.9	88.0 ± 8.0
Female	68.2 ± 5.9	79.7 ± 8.1
Diabetes Mellitus	1,090 (1.4)	5,268 (9.9)
Blood pressure (BP)		
Systolic BP, mmHg	111.0 ± 15.4	123.5 ± 16.9
Diastolic BP, mmHg	69.5 ± 10.1	77.1 ± 11.2
Hypertension		
Grade 1	10,363 (13.3)	13,193 (24.7)
Grade 2	5,672 (7.3)	13,936 (26.1)
Dyslipidemia	8,823 (11.3)	21,308 (39.9)
Triglyceride (TG), mg/dL	80.7 ± 44.2	148.9 ± 94.4
High-density lipoprotein-cholesterol (HDL), mg/dL		
Male	57.1 ± 12.4	49.2 ± 9.7
Female	68.3 ± 14.7	57.3 ± 12.6
Low-density lipoprotein-cholesterol (LDL), mg/dL	106.8 ± 29.4^[3(<0.1)]^	124.1 ± 32.5^[29(0.1)]^
Cholesterol (Chol), mg/dL	187.0 ± 32.8	202.5 ± 36.1
Platelet count, 10^3^/µL	247.4 ± 56.4^[1(<0.1)]^	254.0 ± 57.2^[2(<0.1)]^
Alanine aminotransferase (ALT), U/L	19.7 ± 13.5^[1(<0.1)]^	37.5 ± 28.6^[3(<0.1)]^
Abnormal liver function test	5,953 (7.7)	22,052 (41.3)
Glutamyl Transferase (GGT), U/L	19.4 ± 20.0^[837(1.1)]^	36.8 ± 41.1^[438(0.8)]^
Total bilirubin, mg/dL	1.0 ± 0.4^[38(<0.1)]^	0.9 ± 0.4^[35(<0.1)]^
Albumin, g/dL	4.5 ± 0.2^[41(<0.1)]^	4.5 ± 0.2^[32(<0.1)]^
3-year Average Exposure of Air Pollutant		
PM_2.5_, µg/m^3^	30.3 ± 6.2	31.0 ± 7.0
PM_10_, µg/m^3^	51.1 ± 12.3	52.6 ± 13.9
CO, µg/m^3^	701.8 ± 234.6	707.3 ± 235.2
O_3_, µg/m^3^	52.7 ± 6.4	53.0 ± 6.6
SO_2_, µg/m^3^	10.6 ± 3.7	11.1 ± 4.3
NO_2_, µg/m^3^	37.3 ± 9.6	37.7 ± 9.7
Cardiometabolic criteria of MASLD^a^		
Criteria 1	19,557 (25.0)	46,238 (86.5)
Criteria 2	21,175 (27.1)	33,790 (63.2)
Criteria 3	11,938 (15.3)	23,144 (43.3)
Criteria 4	5,385 (6.9)	21,600 (40.4)
Criteria 5	6,867 (8.8)	13,597 (25.5)

CO: carbon monoxide; MASLD: metabolic dysfunction-associated steatotic liver disease; NO_2_: nitrogen dioxide; O_3_: ozone; PM_2.5_: particulate matter with an aerodynamic diameter ≤2.5 µm; PM_10_: particulate matter with an aerodynamic diameter ≤10 µm; SO_2_: sulfur dioxide; USD: United States dollars

^[]^ Numbers and percentage of cases with missing information for the covariate

^a^MASLD criteria and prevalence according to:

Criteria 1: BMI ≥23 kg/m^2^ (in Asians) or WC >94 cm for men or WC >80 cm for women

Criteria 2: fasting serum glucose ≥100 mg/dL or HbA1c ≥5.7% or type 2 diabetes or under treatment for type 2 diabetes

Criteria 3: BP ≥130/85 mmHg or under specific antihypertensive drug treatment

Criteria 4: plasma TG ≥150 mg/dL or under lipid lowering treatment

Criteria 5: plasma HDL ≤40 mg/dL for men or HDL ≤50 mg/dL for women or under lipid lowering treatment

**Fig. 2:**
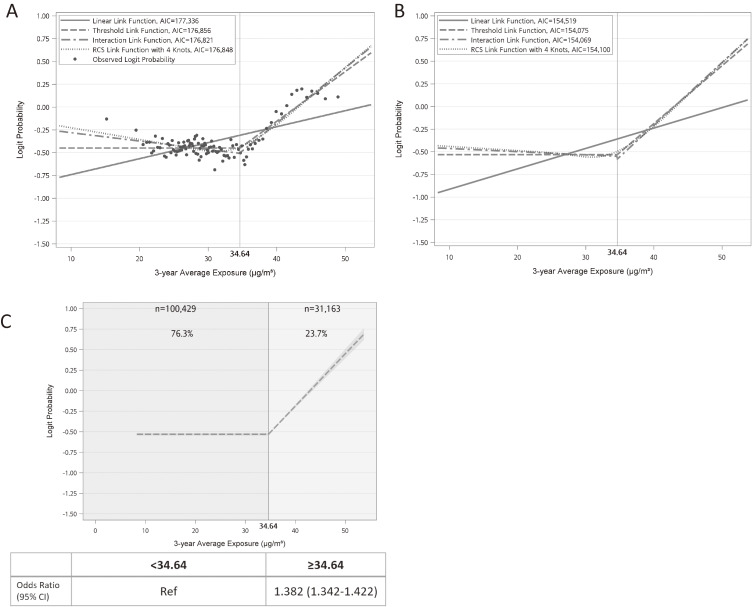
Observed and predicted logit probability of 3-year average PM_2.5_ exposure for MASLD. (A) Scatter plot of the observed logit probability and comparisons of the predicted logit probability by crude models with various link functions: linear (proportional odds) link function, threshold link function, interaction link function, and restricted cubic spline (RCS) link function; (B) Comparisons of the predicted logit probability by covariate adjusted models with various link functions; and (C) The model with “threshold link function” was finally selected to analyze the covariate adjusted logit probability in association with 3-year average exposure of PM_2.5_; and categorization of exposure was based on the threshold level and was used to calculate the covariate adjusted odds ratio and 95% confidence interval (CI) of MASLD. The models in Fig. 2B and 2C were adjusted for age, sex, marriage, education, household income, alcohol, smoking, fried food intake, vegetable intake, fruit intake, sugary drink intake, habit of regular exercise and the year of participant enrollment. MASLD: metabolic dysfunction-associated steatotic liver disease; PM_2.5_: particulate matter with an aerodynamic diameter ≤2.5 µm.

Figure 2 illustrated the process of determining the best fitted link function between PM_2.5_ and MASLD in a logistic regression model. In Fig. 2A, the observed logit probability showed a non-linear relationship with a potential inflection point and the Box-Tidwell test confirmed violation against proportional odds. To determine the inflection point, we fitted two linear link functions below and above a specific cutoff level, then estimated the proper slope of the two linear functions, which finally identified the best-fitted inflection point at 34.64 µg/m^3^. Subsequently, we constructed one-pollutant regression models (PM_2.5_) with each of the 4 link functions: linear, threshold, interaction, and RCS. The model with interaction link function yielded the lowest AIC. However, after adjusting for multiple covariates in one-pollutant model, both interaction and threshold link functions resulted in very similar AIC values (Fig. 2B). According to our predefined rule of link function selection, the model with threshold link function was used to address the relationship between exposure to PM_2.5_ and odds of MASLD (Fig. 2C). For the PM_2.5_ exposure above 34.64 µg/m^3^, each 1 µg/m^3^ increase exhibited an aOR of 1.068 (95% CI: 1.064 to 1.072) for MASLD.

Similar processes were repeated to determine the best-fitted link function for the association of MASLD with PM_10_, O_3_, CO, SO_2_, and NO_2_, respectively; and the results are shown in Fig. S1–S5. The interaction link function was considered as the best-fitted link function for PM_10_ with a cutoff concentration level of 57.93 µg/m^3^ (Fig. S1C) and for CO with cutoff of 643.6 µg/m^3^ (Fig. S3C). Since RCS function with 4 knots showed the better fit for the relationship between O_3_ and MASLD, the RCS functions with 3–7 knots were further compared (Fig. S2B and S2C). As such, the RCS link function with 3 knots was finally considered as the best-fitted link function for O_3_. Through the same process, an RCS link function with 3 knots (Fig. S4D) and 4 knots (Fig. S5D) was finally determined to be the best-fitted link function for SO_2_ and NO_2_, respectively.

**Fig. 3:**
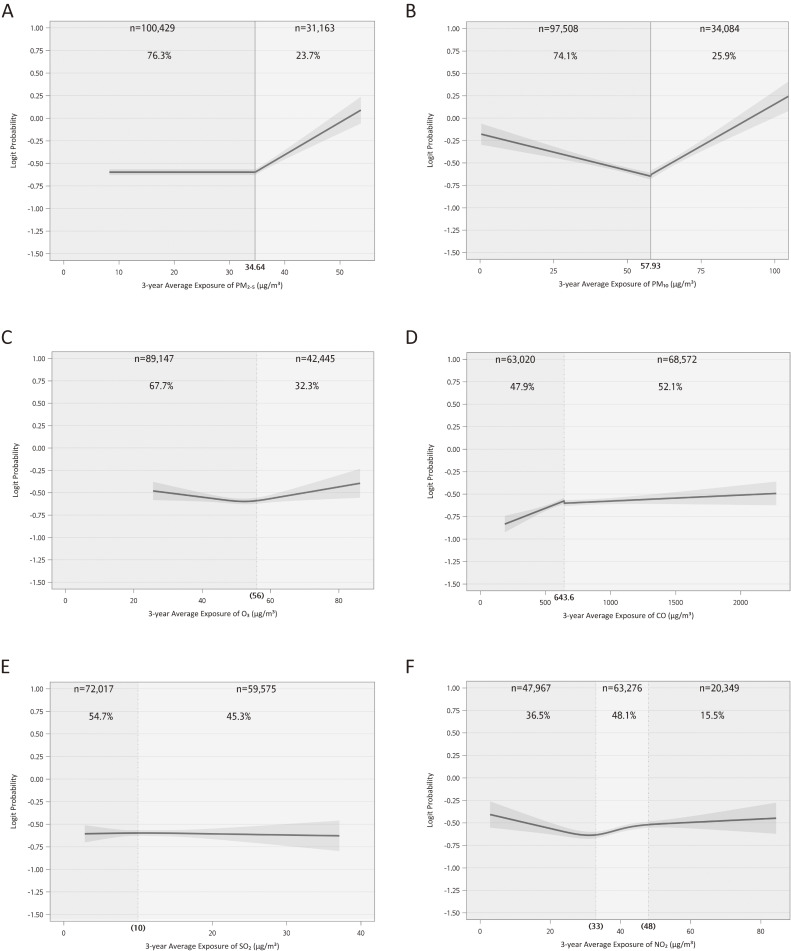
Predicted logit probability from six-pollutant model of 3-year average air pollutant exposure for MASLD. (A) the “threshold link function” for particulate matter with an aerodynamic diameter ≤2.5 µm, PM_2.5_; (B) the “interaction link function” for particular matter with an aerodynamic diameter ≤10 µm, PM_10_; (C) the “restricted cubic spline (RCS) link function” with 3 knots for ozone, O_3_; (D) the “interaction link function” for carbon monoxide, CO; (E) the “RCS link function” with 3 knots for sulfur dioxide, SO_2_; and (F) the “RCS link function” with 4 knots for nitrogen dioxide, NO_2_. The model was adjusted for age, sex, marriage, education, household income, alcohol, smoking, fried food intake, vegetable intake, fruit intake, sugary drink intake, habit of regular exercise and the year of participant enrollment. The plots were generated by adjusting covariates and other pollutants to their mean values. The cut-off values in Fig. 3A, 3B and 3D were the inflection values based on nonlinear regressions, while cut-off values in Fig. 3C, 3E and 3F were based on observation of restricted cubic spline plots. MASLD: Metabolic dysfunction-associated steatotic liver disease.

Both Fig. 3 and Table 2 showed the results from covariate adjusted six-pollutant model. Based on the best-fitted link function, per 1 µg/m^3^ increase in PM_2.5_ exhibited a significantly increased aOR of 1.036 (95% CI: 1.030 to 1.042) for MASLD when exposure level above 34.64 µg/m^3^. On the other hand, a 1 µg/m^3^ increase in PM_10_ was found to be significantly associated with reduced and increased risk of MASLD at different exposure levels. For exposure level below 57.93 µg/m^3^, the aOR was 0.992 (95% CI: 0.990–0.993), while for exposure level above 57.93 µg/m^3^, the aOR was 1.019 (95% CI: 1.016–1.021). The model with interaction link function for CO exhibited an increase and plateau pattern. CO increase per 1 µg/m^3^ was significantly and positively associated with increased aOR of MASLD at CO <643.6 µg/m^3^.

**Table 2:** Covariate adjusted odds ratios of MASLD in relation to 3-year average exposure to various air pollutants estimated from covariate adjusted six-pollutant logistic regression model with various link functions

**Pollutant**	**Exposure range^a^**	**Model 1** **Model based on the best-fitted ** **link function for each pollutant** **AIC: 153,539**	**Model 2** **Model with categorical ** **exposure based on the cut-off ** **points determined by Model 1** **AIC: 154,115**

		**Adjusted OR (95% CI) ** **per 1 µg/m^3^ increase^b^**	**Adjusted OR (95% CI)^bc^**
PM_2.5_		**Threshold link function**	
	<34.64 µg/m^3^	1.000 (1.000–1.000)	Ref
	≥34.64 µg/m^3^	1.036 (1.030–1.042)	1.235 (1.190–1.281)
PM_10_		**Interaction link function**	
	<57.93 µg/m^3^	0.992 (0.990–0.993)	Ref
	≥57.93 µg/m^3^	1.019 (1.016–1.021)	1.152 (1.110–1.196)
O_3_		**3-knot RCS link function**	
	<56 µg/m^3^	0.996 (0.994–0.999)	Ref
	≥56 µg/m^3^	1.006 (1.002–1.011)	1.074 (1.043–1.107)
CO		**Interaction link function**	
	<643.6 µg/m^3^	1.0006 (1.0004–1.0007)	Ref
	≥643.6 µg/m^3^	1.0001 (1.0000–1.0001)	1.127 (1.095–1.161)
SO_2_		**3-knot RCS link function**	
	<10 µg/m^3^	1.001 (0.992–1.010)	Ref
	≥10 µg/m^3^	0.999 (0.994–1.004)	1.061 (1.031–1.091)
NO_2_		**4-knot RCS link function**	
	<33 µg/m^3^	0.992 (0.989–0.996)	Ref
	33–48 µg/m^3^	1.008 (1.008–1.008)	1.043 (1.012–1.074)
	≥48 µg/m^3^	1.002 (0.998–1.006)	1.142 (1.094–1.192)

AIC: Akaike information criterion; CI: confidence interval; CO: carbon monoxide; MASLD: metabolic dysfunction-associated steatotic liver disease; NO_2_: nitrogen dioxide; O_3_: ozone; PM_2.5_: particulate matter with an aerodynamic diameter ≤2.5 µm; PM_10_: particulate matter with an aerodynamic diameter ≤10 µm; OR: odds ratio; RCS: restricted cubic spline; SO_2_: sulfur dioxide

^a^3-year average of exposure of pollutants

^b^Adjusted for age, sex, marriage, education, household income, alcohol, smoking, fried food intake, vegetable intake, fruit intake, sugary drink intake, habit of regular exercise and the year of participant enrollment.

^c^The cut-off values for PM_2.5_, PM_10_ and CO were the inflection values based on nonlinear regressions, while cut-off values for O_3_, SO_2_ and NO_2_ were based on observation of restricted cubic spline plots.

The logistic regression model with RCS link function for O_3_ showed a tendency of slight fluctuation followed by an increase across the exposure range. One µg/m^3^ increase in O_3_ was significantly associated with an increased aOR of MASLD at 1.006 (95% CI: 1.002–1.011) only when O_3_ ≥56 µg/m^3^. SO_2_ was the sole pollutant demonstrating no association with MASLD, as its aOR did not significantly differ from 0 across the entire exposure range. The link function for NO_2_ showed a trend of decrease, rising and reaching a plateau in aOR of MASLD over the exposure range. For NO_2_ exposure in the range of 33–48 µg/m^3^, per 1 µg/m^3^ increase was associated with a significantly elevated aOR of MASLD at 1.008 (95% CI: 1.008–1.008) (Table 2).

The aORs of MASLD estimated from the six-pollutant model in association with air pollutant exposure categorized by the inflection points based on the best-fitted link functions are also shown in the right column of Table 2. In this model, compared to participants with exposure levels below the inflection points, PM_2.5_, PM_10_, O_3_, CO and NO_2_ all exhibited significantly increased aOR above the inflection points, while SO_2_ categorizations did not.

Table S2 displays aORs of MASLD in association with 1 µg/m^3^ increase in air pollutant estimated from logistic regression model with linear link functions assumed for all air pollutants. In this model, all pollutants, except for PM_10_, demonstrated significant increase in aORs of MASLD in association with 1 µg/m^3^ increase in exposure. Comparatively, the best fitted model (Fig. 3 and left column of Table 2) exhibited better model fitting (AIC: 153,539) than the regression model with categorized exposure (right column of Table 2) (AIC: 154,115) and the regression model with linear link functions assumed (Table S2) (AIC: 154,074), respectively. Due to marginally high correlation between PM_2.5_ and PM_10_ (Table S3), A 5-pollutant model without PM_10_ was built. As shown in Table S4 and Fig. S6, the associations between air pollutants and MASLD remained consistent with the main model, with the exception of SO_2_, which displayed a diminished J-shaped relationship.

## Discussions

Although the nature of cross-sectional design precludes the causal inference, the current study demonstrated a non-linear relationship between 3-year exposure and MASLD based on a large-scale sample of 131,592 health checkup participants. After adjustment for potential confounders, a “threshold link function”, “interaction link functions” and “restricted cubic spline (RCS) link functions” showed the best-fitted associations of MASLD with PM_2.5_, PM_10_/CO, and O_3_ /SO_2_/NO_2_. The significantly positive associations between air pollution and MASLD were only found above (PM_2.5_, PM_10_, O_3_), below (CO), and within (NO_2_) certain levels of exposure in a six-pollutant model. While the results from two sensitivity analyses that excluded PM_10_ from the model to avoid potential problem of co-linearity or assumed a linear link functions between all air pollutants and MASLD yielded the highly comparable results. Besides, the non-linear model also exhibited a superior ability to predict probability (odds) of MASLD (Fig. 3 and Table 2), compared to the models based on categorized exposure or assumed linear link functions.

In this study, 40.6% of the health checkup participants were identified to have MASLD. The prevalence of NAFLD in Taiwan in meta-analysis based on published data was estimated to be 33.29% (26.42%–40.96%) [37] and it might exceed 40% in health checkup participants [38]. In prior studies, VoPham et al. analyzed the US National Inpatient Sample using a spatiotemporal exposure model based on ZIP codes, revealing an OR of 1.24 (95% CI, 1.15–1.33) for NAFLD per 10 µg/m^3^ increase of PM_2.5_ [19]. Guo et al. investigated the Chinese CMEC cohort, utilizing a satellite-based forest approach for exposure assessment, showing associations with 3-year average PM_2.5_ and NO_2_ exposure, resulting in ORs of 1.29 (1.25–1.34) and 1.15 (1.12–1.17) for MAFLD per 10 µg/m^3^ increment, respectively [17]. Sun et al. utilized the Taiwan MJ cohort and employed satellite-based atmospheric optical depth measurement for exposure assessment, reporting hazard ratios of 1.06 (1.04–1.09) and 1.05 (1.03–1.07) for NAFLD, when PM_2.5_ increased every 1 µg/m^3^ above 23.5 µg/m^3^, dependent on the determinant for fatty liver status [18]. Li et al. utilized UK Biobank data and a land-use regression model for exposure assessment, and found hazard ratios of 1.10 (1.05–1.14), 1.14 (1.09–1.20) and 1.19 (1.13–1.24) for PM_2.5_, PM_10_ and NO_2_, respectively, for each interquartile range increase [20]. Our current study using Taiwan MJ cohort and land-use regression in combination with machine learning algorithm for exposure assessment. Similar to most of the abovementioned studies, our data exhibited positive associations of 3-year elevated exposure to PM_2.5_, PM_10_ and NO_2_ with MASLD. However, our study advanced the knowledge by demonstrating that the relationships between the above air pollutants and MASLD were not linear, which was implicitly suggested in the studies by Guo and Sun [17, 18], who nonetheless did not further elaborate such potential non-linear association between air pollution exposure and fatty liver disease.

A recent study demonstrated that long term exposure to ambient PMs and NO_2_ is associated with both fatty liver disease and liver cirrhosis [39]. However, commonly monitored pollutants like SO_2_ and CO on hepatic injury or fatty liver diseases has not been explored in previous research. It is worth noting that air pollutants rarely existed in a single-pollutant form [40], and the typical pollutants used for air quality assessment, as the 6 pollutants in our study, were intercorrelated (e.g., NO_2_ is a precursor of O_3_). Considering multipollutant exposures collectively is crucial for a comprehensive understanding of the impact of air pollution, especially from public health regulation perspectives [41]. The dose-response relationship between a specific air pollutant and health outcomes may change when accounting for other pollutants, as observed in our study. Simultaneous inclusion of the six air pollutants in our regression model adds further reassurance of validity for the associations between PM_2.5_, PM_10_, CO, O_3_, NO_2_ and MASLD. Expanding the MASLD prediction model to encompass a broader spectrum of pollutants could enhance its accuracy. However, regular monitoring of additional pollutants beyond the six pollutants would substantially elevate costs. While incorporating interaction terms could refine the model’s performance, the methodologies for statistical testing are still evolving, and the resulting models may become overly complex, leading to unstable coefficient estimates and challenging interpretation [40].

The mechanism underlying the association between air pollution and MASLD remains unclear. However, animal studies demonstrated that exposure to PM_2.5_ increased hepatic dyslipidemia and increased oxidative stress, resulting in hepatic inflammation similar to non-alcoholic steatohepatitis and fibrosis [42, 43]. On the other hand, inhaled O_3_ was linked to glycolysis dysfunction and glucose intolerance, which may be precursors of metabolic dysfunctions [44]. Chronic exposure to ambient air pollution (particularly PM_2.5_) was associated with metabolic syndrome, systemic and hepatic inflammation in epidemiological studies [14, 45]. Nitrogen dioxide as part of traffic related air pollution was also found to be related to elevated cytokeratin-18 and may be related to liver injury [13]. Oxidative stress and systemic inflammation induced by ambient air pollution may be the major mechanism leading to the presentation of MASLD [46–48].

In the six-pollutant model, as anticipated, some health hazards observed in one-pollutant models were attenuated by the presence of other pollutants, resulting in lower ORs. Sulfur dioxide was the only air pollutant with non-positive association within the whole exposure range. In one pollutant model, SO_2_ exhibited a J-shaped curve (Fig. S4D) but transformed into a null pattern in the six-pollutant model (Fig. 3E). Upon removing PM_10_ from the model, the curve exhibited a more flattened J-shaped association. (Fig. S6D). These findings indicate that SO_2_-MASLD association might be highly influenced by the representativeness of our sample and the SO_2_’s association to other pollutants. Firstly, petrochemical parks and factories in Taiwan are predominantly located in rural, lower socioeconomic areas, whereas MJ clinics are in urban areas. This geographic disparity may restrict the inclusion of individuals from rural, lower economic areas near petrochemical parks, who are likely to have high SO_2_ exposure, in our study. Given the positive correlation between lower socioeconomic status and fatty liver disease [49], enrolling participants solely from MJ clinics might introduce selection bias, underrepresenting those with higher SO_2_ exposure and fatty liver disease. Secondly, since SO_2_ emission primarily originates from industrial sources, its effects might be confounded by other pollutants like PM_2.5_ or NO_2_. This could explain the altered association observed in multipollutant models. Consequently, future research with more precise SO_2_ measurement in regions heavily impacted by petrochemical pollution is essential for a clearer understanding of these associations.

Several strengths were involved in this study. Firstly, it revealed a non-linear correlation between air pollution and MASLD, enabling precise risk estimation in distinct exposure concentration ranges, rather than relying solely on single estimates. Secondly, our exposure assessment method offered superior 50-m × 50-m grid resolution for air pollutant exposure assessment, with enhanced accuracy compared to prior studies. Thirdly, we devised a process to assess the non-linear relationship between chronic air-pollution exposure and MASLD, which was considered essential for understanding air pollution’s health impacts [50, 51]. Moreover, our multipollutant model, integrating all six common pollutants, outperformed regression models with categorized or linear exposure assumptions. By combining the multipollutant non-linear model and exposure assessment approaches in this study, the relationship between air pollution and MASLD can be better illustrated. To the best of our knowledge, this is the first study of its kind in the literature. This improved methodology may advance the exploration of mechanisms linking air pollution and MASLD.

Despite the aforementioned strengths, several limitations warrant consideration. Firstly, air pollution exposure assessment based on residential addresses may introduce exposure misclassification. Although we combined land-use regression and machine learning for exposure assessment, there precision of these measures may still be subject to variability, potentially underestimation air pollution-MASLD associations, aligning with previous published studies [17–20]. Secondly, this cross-sectional study with retrospective exposure assessment lacks disease course information before checkup. The cross-sectional design and absence of a temporal component preclude the establishment of causality in this study. Thirdly, comorbidity ascertainment via self-report also carries a risk of misclassification. Incomplete adjustment for potential confounders, like DM and HTN, could lead to residual confounding and overestimation the impact of air pollution on MASLD. Furthermore, despite our rigorous attempts to adjust for confounding variables, unaccounted factors such as genetic predispositions or occupational exposures may persist in this study. Fourthly, the six-pollutant model included both PM_2.5_ and PM_10_, and their potential collinearity might affect this model. The naive Pearson’s correlation analysis showed a coefficient of r = 0.770 (0.768–0.772) between PM_2.5_ and PM_10_, and r = 0.677 (0.674–0.680) between PM_10_ and SO_2_ (Table S3), which is marginally acceptable. The tolerance was ≥0.27 (with PM_10_ having the lowest tolerance), and the variance inflation factors (VIF) was ≤3.69 (with PM_10_ having the largest VIF) for all 6 pollutants and covariates indicating that multicollinearity should not be a problematic issue. A sensitivity analysis with 5-pollutant model excluding PM_10_ yielded a very similar results (Table S4 and Fig. S6), supporting the validity of our findings. Finally, our results, derived from a health-checkup cohort in Taiwan, warrant validation in diverse populations with varying environmental exposures, genetic predispositions, and lifestyle patterns.

Our study identified non-linear thresholds for air pollutant associations with MASLD, with some inflection points exceeding the World Health Organization (WHO) recommended annual air quality levels [52]. For example, 34.64 µg/m^3^ compared to WHO’s 5 µg/m^3^ for PM_2.5_, or 33 µg/m^3^ compared to WHO’s 5 µg/m^3^ for NO_2_. Notably, 23.7% of our participant cases fell within the effective exposure range for PM_2.5_ and 48.1% for NO_2_. These findings highlight the necessity of mitigating air pollution exposure as a complementary strategy to conventional diet, behavior, and metabolic risk factor management in preventing MASLD.

## Conclusions

In conclusion, this large-scale cross-sectional study demonstrated non-linear associations between 3-year exposure to six common air pollutants and MASLD at time of health check-up, in which PM_2.5_, PM_10_, O_3_, CO and NO_2_ were positively associated with MASLD only at certain ranges of concentrations.

## Abbreviations

AIC: Akaike information criterion
ALT: alanine aminotransferase
BMI: body mass index
Chol: cholesterol
CIs: confidence intervals
CO: carbon monoxide
DM: Diabetes mellitus
HTN: Hypertension
HDL: high-density lipoprotein-cholesterol
MASLD: metabolic dysfunction-associated fatty liver disease
NAFLD: Non-alcoholic fatty liver disease
NO_2_: nitrogen dioxide
OR: odds ratio
O_3_: ozone
PM_2.5_: particulate matter with an aerodynamic diameter ≤2.5 µm
PM_10_: particulate matter with an aerodynamic diameter ≤10 µm
RCS: Restricted cubic spline
SO_2_: sulfur dioxide
TG: triglyceride
WHO: World Health Organization

## Supplementary information

**Additional file 1:** **Table S1.** Air pollution exposure concentration averaged over different lengths of years. **Table S2.** Covariates adjusted odds ratios of MASLD in association with exposure to various air pollutants in the six-pollutant logistic regression model with linear (proportional odds) link functions. (Akaike information criterion, AIC: 154,074). **Table S3.** Pearson correlation coefficients between each pair of the six air pollutants. **Table S4.** Covariate adjusted odds ratios of MASLD in relation to various air pollutants estimated from the multivariate 5-pollutant logistic regression model with various best-fitted link functions. **Fig. S1** Observed and predicted logit probability in association with 3-year average of particulates matter with aerodynamic diameter ≤ 10 µm (PM_10_) exposure for metabolic dysfunction-associated steatotic liver disease (MASLD). **Fig. S2** Observed and predicted logit probability in association with 3-year average of ozone (O_3_) exposure for metabolic dysfunction-associated steatotic liver disease (MASLD). **Fig. S3** Observed and predicted logit probability in association with 3-year average of carbon monoxide (CO) exposure for metabolic dysfunction-associated steatotic liver disease (MASLD). **Fig. S4** Observed and predicted logit probability in association with 3-year average of sulfur dioxide (SO_2_) exposure for metabolic dysfunction-associated steatotic liver disease (MASLD). **Fig. S5** Observed and predicted logit probability in association with 3-year average of nitrogen dioxide (NO_2_) exposure for metabolic dysfunction-associated steatotic liver disease (MASLD). **Fig. S6** Logit probability predicted from covariates adjusted five-pollutant model in association with 3-year average exposure to 5 air pollutants for metabolic dysfunction-associated steatotic liver disease (MASLD).

## Declarations

### Data availability statement

The data utilized for this study are not publicly available. However, de-identified data may be available on request, subject to approval by the internal review board and under a formal data use agreement. Contact the corresponding author for more information.
