# Supplementary material for: Non-linear association between long-term air pollution exposure and risk of metabolic dysfunction-associated steatotic liver disease
Source: Environ Health Prev Med. 2024 Feb 10;29:7. doi: 10.1265/ehpm.23-00271 (PMC10898959; doi:10.1265/ehpm.23-00271)
Supplement: Supplementary file 1 — Additional file 1: Table S1. Air pollution exposure concentration averaged over different lengths of years. Table S2. Covariates adjusted odds ratios of MASLD in association with exposure to various air pollutants in the six-pollutant logistic regression model with linear (proportional odds) link functions. (Akaike information criterion, AIC: 154,074). Table S3. Pearson correlation coefficients between each pair of the six air pollutants. Table S4. Covariate adjusted odds ratios of MASLD in relation to various air pollutants estimated from the multivariate 5-pollutant logistic regression model with various best-fitted link functions. Fig. S1 Observed and predicted logit probability in association with 3-year average of particulates matter with aerodynamic diameter ≤ 10 µm (PM10) exposure for metabolic dysfunction-associated steatotic liver disease (MASLD). Fig. S2 Observed and predicted logit probability in association with 3-year average of ozone (O3) exposure for metabolic dysfunction-associated steatotic liver disease (MASLD). Fig. S3 Observed and predicted logit probability in association with 3-year average of carbon monoxide (CO) exposure for metabolic dysfunction-associated steatotic liver disease (MASLD). Fig. S4 Observed and predicted logit probability in association with 3-year average of sulfur dioxide (SO2) exposure for metabolic dysfunction-associated steatotic liver disease (MASLD). Fig. S5 Observed and predicted logit probability in association with 3-year average of nitrogen dioxide (NO2) exposure for metabolic dysfunction-associated steatotic liver disease (MASLD). Fig. S6 Logit probability predicted from covariates adjusted five-pollutant model in association with 3-year average exposure to 5 air pollutants for metabolic dysfunction-associated steatotic liver disease (MASLD). [file ehpm-29-007-s001.docx]

**Supplementary Tables and Figures**

**Table S1.** Air pollution exposure concentration averaged over different lengths of years

|  |  | 1 year | 2 years | 3 years | 4 years |
| --- | --- | --- | --- | --- | --- |
| PM_2.5_   (μg/m^3^) | Mean ± SD | 29.6 ± 6.4 | 30.0 ± 6.4 | 30.5 ± 6.5 | 31.0 ± 6.5 |
|  | Median (IQR) | 28.7 (7.8) | 29.0 (7.9) | 29.4 (7.9) | 29.9 (8.0) |
|  | Minimum | 6.8 | 7.6 | 8.0 | 8.0 |
|  | Maximum | 53.9 | 53.0 | 53.8 | 53.6 |
| PM_10_   (μg/m^3^) | Mean ± SD | 50.0 ± 13.2 | 50.6 ± 13.0 | 51.4 ± 12.9 | 52.0 ± 12.8 |
|  | Median (IQR) | 47.6 (15.4) | 47.7 (14.9) | 48.5 (14.8) | 49.2 (14.6) |
|  | Minimum | 0.3 | 0.5 | 0.4 | 0.5 |
|  | Maximum | 110.6 | 108.2 | 107.6 | 107.4 |
| O_3_   (μg/m^3^) | Mean ± SD | 52.0 ± 6.7 | 52.4 ± 6.6 | 52.6 ± 6.5 | 52.7 ± 6.6 |
|  | Median (IQR) | 52.3 (9.0) | 52.7 (8.8) | 52.8 (8.7) | 52.8 (8.7) |
|  | Minimum | 23.6 | 25.3 | 25.1 | 25.4 |
|  | Maximum | 88.4 | 86.7 | 86.6 | 86.3 |
| CO  (μg/m^3^) | Mean ± SD | 704.1 ± 236.9 | 705.6 ± 235.4 | 711.3 ± 236.2 | 716.8 ± 234.7 |
|  | Median (IQR) | 658.2 (280.7) | 658.7 (273.6) | 663.4 (271.5) | 670.8 (274.3) |
|  | Minimum | 178.4 | 188.0 | 187.5 | 189.3 |
|  | Maximum | 2284.9 | 2297.7 | 2277.8 | 2262.4 |
| SO_2_   (μg/m^3^) | Mean ± SD | 10.2 ± 3.8 | 10.4 ± 3.9 | 10.7 ± 3.9 | 11.0 ± 4.0 |
|  | Median (IQR) | 9.2 (3.9) | 9.3 (4.0) | 9.6 (4.1) | 9.9 (4.2) |
|  | Minimum | 2.7 | 2.8 | 2.8 | 2.8 |
|  | Maximum | 36.0 | 36.0 | 36.0 | 37.5 |
| NO_2_   (μg/m^3^) | Mean ± SD | 37.1 ± 9.8 | 37.2 ± 9.8 | 37.6 ± 9.8 | 38.1 ± 9.8 |
|  | Median (IQR) | 36.4 (14.0) | 36.6 (14.1) | 36.9 (14.2) | 37.4 (14.1) |
|  | Minimum | 2.7 | 2.8 | 2.9 | 2.9 |
|  | Maximum | 85.5 | 84.4 | 84.4 | 85.8 |

CO: carbon monoxide; IQR: interquartile range; NO_2_: nitrogen dioxide; O_3_: ozone; PM_2.5_: particulate matter with an aerodynamic diameter ≤ 2.5μm; PM_10_: particulate matter with an aerodynamic diameter ≤ 10μm; SD: standard deviation; SO_2_: sulfur dioxide.**Table S2.** Covariates adjusted odds ratios of MASLD in association with exposure to various air pollutants in the six-pollutant logistic regression model with linear (proportional odds) link functions. (Akaike information criterion, AIC: 154,074)

| Air pollutants  per 1μg/m^3^ increase | | Covariate adjusted odds ratio ^a^  (95% confidence interval) |
| --- | --- | --- |
| PM_2.5_ |  | 1.015 (1.012-1.018) |
| PM_10_ |  | 0.999 (0.997-1.001) |
| O_3_ |  | 1.006 (1.004-1.009) |
| CO |  | 1.0001 (1.0001-1.0002) |
| SO_2_ |  | 1.016 (1.012-1.021) |
| NO_2_ |  | 1.003 (1.001-1.005) |

CO: carbon monoxide; MASLD: metabolic dysfunction-associated steatotic liver disease; NO_2_: nitrogen dioxide; O_3_: ozone; PM_2.5_: particulate matter with an aerodynamic diameter ≤ 2.5μm; PM_10_: particulate matter with an aerodynamic diameter ≤ 10μm; SO_2_: sulfur dioxide

^a^ Covariates adjusted included age, sex, marriage, education, household income, alcohol, smoking, fried food intake, vegetable intake, fruit intake, sugary drink intake, habit of regular exercise and the year of participant enrollment.

**Table S3.** Pearson correlation coefficients between each pair of the six air pollutants

|  | PM_2.5_ | PM_10_ | O_3_ | CO | SO_2_ | NO_2_ |
| --- | --- | --- | --- | --- | --- | --- |
| PM_2.5_ | 1.00 |  |  |  |  |  |
| PM_10_ | 0.770  (0.768-0.772) | 1.00 |  |  |  |  |
| O_3_ | 0.048  (0.043-0.054) | 0.198  (0.193-0.203) | 1.00 |  |  |  |
| CO | 0.137  (0.131-0.142) | -0.014  (-0.019 - -0.008) | -0.539  (-0.543 - -0.535) | 1.00 |  |  |
| SO_2_ | 0.499  (0.495-0.503) | 0.677  (0.674-0.680) | 0.093  (0.088-0.098) | 0.028  (0.022-0.033) | 1.00 |  |
| NO_2_ | 0.075  (0.070-0.080) | 0.135  (0.130-0.141) | -0.450  (-0.454 - -0.446) | 0.477  (0.473-0.482) | 0.377  (0.372-0.381) | 1.00 |

CO: carbon monoxide; NO_2_: nitrogen dioxide; O_3_: ozone; PM_2.5_: particulate matter with an aerodynamic diameter ≤ 2.5μm; PM_10_: particulate matter with an aerodynamic diameter ≤ 10μm; SO_2_: sulfur dioxide

**Table S4.** Covariate adjusted odds ratios of MASLD in relation to various air pollutants estimated from the multivariate 5-pollutant logistic regression model with various best-fitted link functions

| Pollutant | Exposure range ^a^ | **Model 1**  **Model based on the best-fitted link function for each pollutant** ^b^ AIC: 153,678 | **Model 2**  **Model with categorical exposure by the cut-off points in Model 1** ^bc^ AIC: 154,169 | **Model 3**  **Model with linear link functions** ^b^  AIC: 154,074 |
| --- | --- | --- | --- | --- |
|  |  | Adjusted OR (95% CI) per 1μg/m^3^ increase | Adjusted OR (95% CI) | Adjusted OR (95% CI) per 1μg/m^3^ increase |
| PM_2.5_ | <34.64μg/m^3^  ≥34.64μg/m^3^ | **Threshold link function**  1.000 (1.000-1.000)  1.058 (1.053-1.062) | Ref  1.340 (1.301-1.381) | 1.018 (1.015-1.020) |
| O_3_ | <56μg/m^3^  ≥56μg/m^3^ | **3-knot RCS link function**  0.997 (0.995-0.999)  1.011 (1.007-1.015) | Ref  1.090 (1.059-1.123) | 1.007 (1.005-1.009) |
| CO | <643.6μg/m^3^  ≥643.6μg/m^3^ | **Interaction link function**  1.0006 (1.0005-1.0007)  1.0001 (0.9999-1.0001) | Ref  1.120 (1.088-1.153) | 1.0002  (1.0001-1.0003) |
| SO_2_ | <10μg/m^3^  ≥10μg/m^3^ | **3-knot RCS link function**  0.983 (0.976-0.989)  1.007 (1.002-1.012) | Ref  1.090 (1.061-1.121) | 1.015 (1.011-1.019) |
| NO_2_ | <33μg/m^3^  33-48μg/m^3^  ≥48μg/m^3^ | **4-knot RCS link function**  0.993 (0.996-0.989)  1.009 (1.009-1.009)  1.001 (0.997-1.005) | Ref  1.050 (1.019-1.081)  1.144 (1.095-1.194) | 1.004 (1.002-1.006) |

AIC: Akaike information criterion; CI: confidence interval; CO: carbon monoxide; MASLD: metabolic dysfunction-associated steatotic liver disease; NO_2_: nitrogen dioxide; O_3_: ozone; PM_2.5_: particulate matter with an aerodynamic diameter ≤ 2.5μm; PM_10_: particulate matter with an aerodynamic diameter ≤ 10μm; OR: odds ratio; RCS: restricted cubic spline; SO_2_: sulfur dioxide

^a^ 3-year average of exposure of pollutants

^b^ Adjusted for age, sex, marriage, education, household income, alcohol, smoking, fried food intake, vegetable intake, fruit intake, sugary drink intake, habit of regular exercise and the year of participant enrollment.

^c^ The cut-off values for PM_2.5_ and CO was the inflection value based on nonlinear regression, while cut-off values for O_3_, SO_2_ and NO_2_ were based on observation of restricted cubic spline plots.


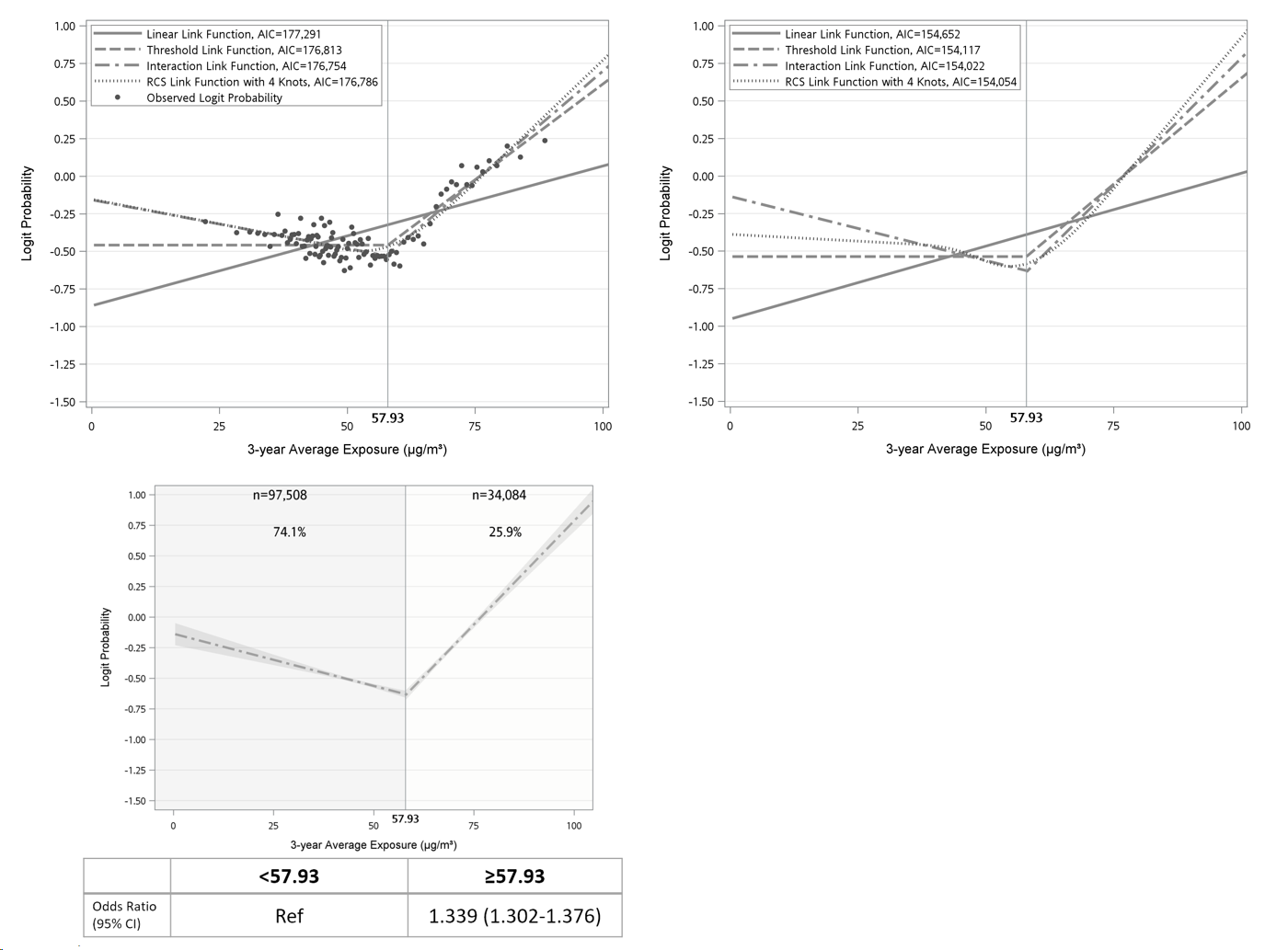


A B

C

**Fig. S1** Observed and predicted logit probability in association with 3-year average of particulates matter with aerodynamic diameter ≤ 10μm (PM_10_) exposure for metabolic dysfunction-associated steatotic liver disease (MASLD): (A) Scatter plot of the observed logit probability and comparisons of the predicted logit probability by crude models with various link functions: linear (proportional odds) link function; threshold link function; interaction link function; and restricted cubic spline (RCS) link function; (B) Comparisons of predicted logit probability by covariate adjusted models with various link functions; and (C) The model with “interaction link function” was finally selected to analyze the covariate adjusted logit probability in association with 3-year average exposure of PM_10_; and categorization of exposure was determined using the inflection point in the calculation of the covariate adjusted odds ratio and 95% confidence interval (CI) of MASLD. The models in **Fig. S1B** and **S1C** were adjusted for age, sex, marriage, education, household income, alcohol, smoking, fried food intake, vegetable intake, fruit intake, sugary drink intake, habit of regular exercise and the year of participant enrollment..


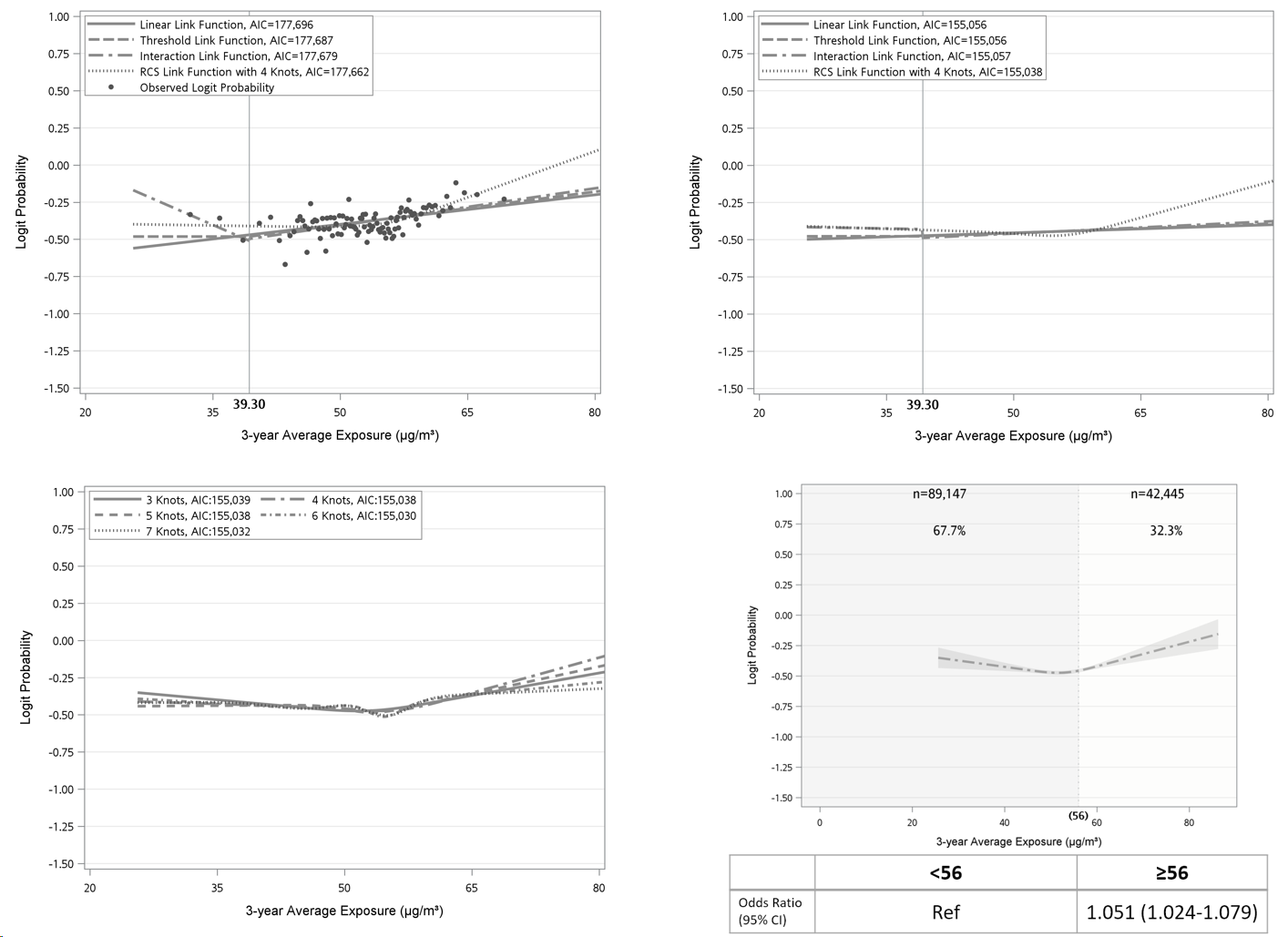


A B

C D

**Fig. S2** Observed and predicted logit probability in association with 3-year average of ozone (O_3_) exposure for metabolic dysfunction-associated steatotic liver disease (MASLD): (A) Scatter plot of the observed logit probability and comparisons of the predicted logit probability by various crude models: linear (proportional odds) link function; threshold link function; interaction link function; and restricted cubic spline (RCS) link function; (B) Comparisons of predicted logit probability by covariate adjusted models with various link functions; (C) Comparisons of predicted logit probability by covariate adjusted models of RCS link functions of 3 to 7 knots; and (D) The model with “3-knot RCS link function” was finally selected to analyze the covariate adjusted logit probability in association with 3-year average exposure of O_3_; and categorization of exposure was determined using cut-off value based on the curve of the graph in the calculation of the covariate adjusted odds ratios and 95% confidence interval (CI) of MASLD. The models in **Fig. S2B, S2C** and **S2D** were adjusted for age, sex, marriage, education, household income, alcohol, smoking, fried food intake, vegetable intake, fruit intake, sugary drink intake, habit of regular exercise, and the year of participant enrollment.


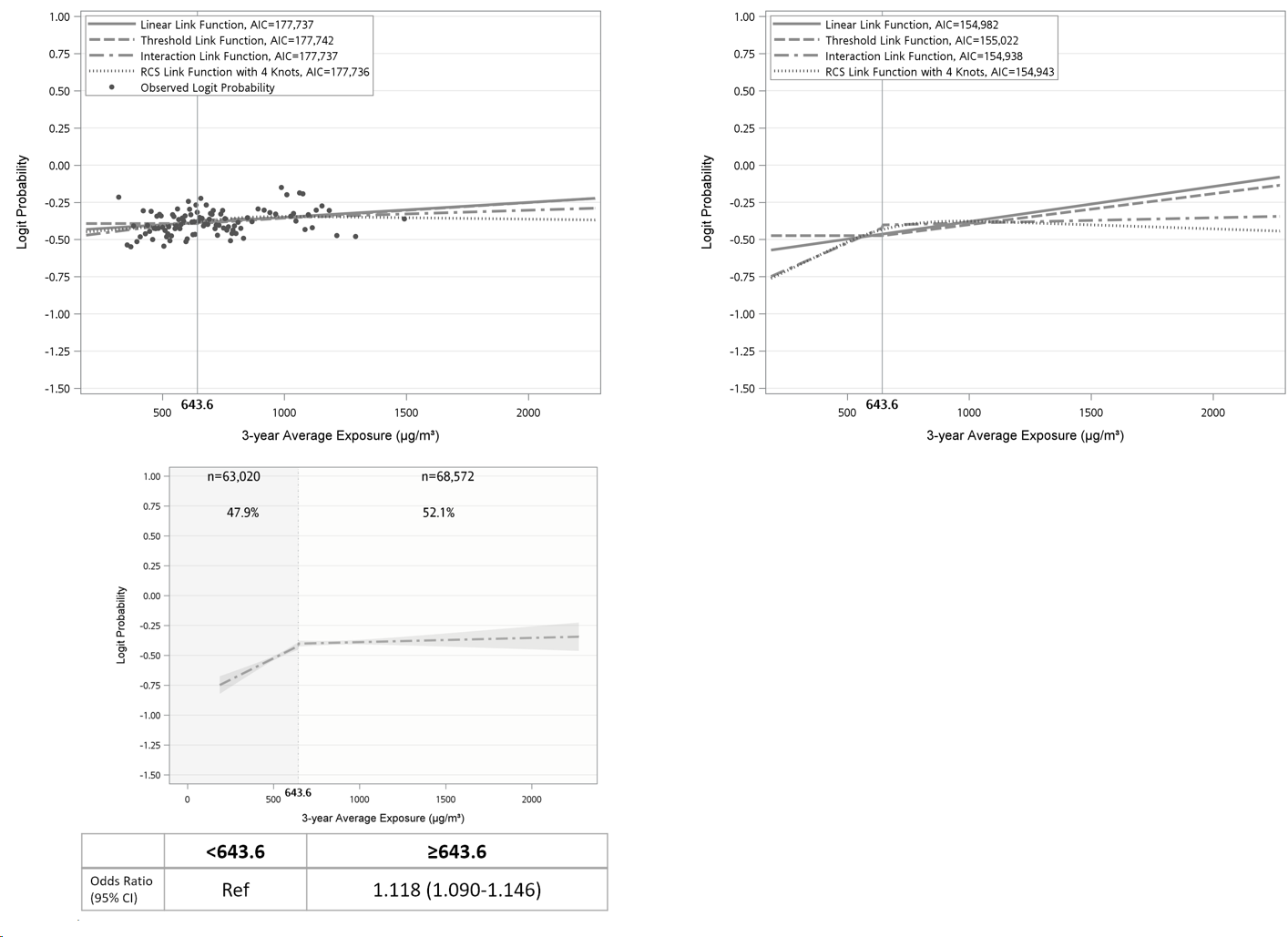


C

A B

**Fig. S3** Observed and predicted logit probability in association with 3-year average of carbon monoxide (CO) exposure for metabolic dysfunction-associated steatotic liver disease (MASLD): (A) Scatter plot of the observed logit probability and comparisons of predicted logit probability by various crude models: linear (proportional odds) link function; threshold function; interaction link function; and restricted cubic spline curve (RCS) link function; (B) Comparisons of predicted logit probability by covariate adjusted models with various link functions; (C) The model with “interaction link function” was finally selected to analyze covariate adjusted logit probability in association with 3-year average exposure of CO ; and categorization of exposure was determined using cut-off value based on the curve of the graph in the calculation of the covariate adjusted odds ratios and 95% confidence interval (CI) of MASLD. The models in **Fig. S3B, S3C** were adjusted for age, sex, marriage, education, household income, alcohol, smoking, fried food intake, vegetable intake, fruit intake, sugary drink intake, habit of regular exercise and the year of participant enrollment.


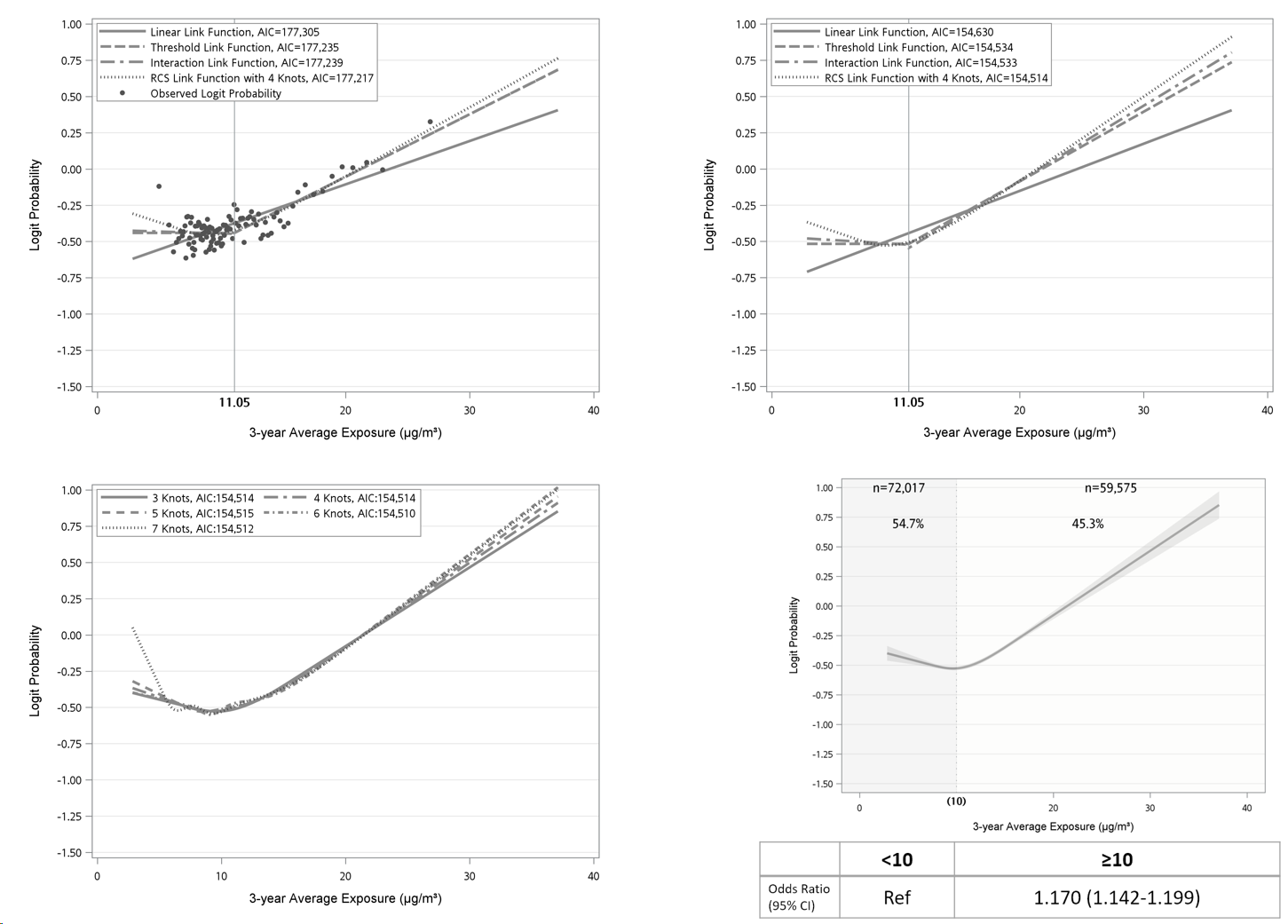


C D

A B

**Fig. S4** Observed and predicted logit probability in association with 3-year average of sulfur dioxide (SO_2_) exposure for metabolic dysfunction-associated steatotic liver disease (MASLD): (A) Scatter plot of the observed logit probability and comparisons of the predicted logit probability by various crude models: linear (proportional odds) link function; threshold link function; interaction link function; and restricted cubic spline (RCS) link function; (B) Comparisons of predicted logit probability by covariate adjusted models with various link functions; (C) Comparisons of predicted logit probability by covariate adjusted models of RCS link functions of 3 to 7 knots; and (D) The model with “3-knot RCS link function” was finally selected to analyze covariate adjusted logit probability in association with 3-year average exposure of SO_2_ ; and categorization of exposure was determined using cut-off value based on the curve of the graph in the calculation of the covariate adjusted odds ratios and 95% confidence interval (CI) of MASLD. The models in **Fig. S4B, S4C** and **S4D** were adjusted for age, sex, marriage, education, household income, alcohol, smoking, fried food intake, vegetable intake, fruit intake, sugary drink intake, habit of regular exercise and the year of participant enrollment.


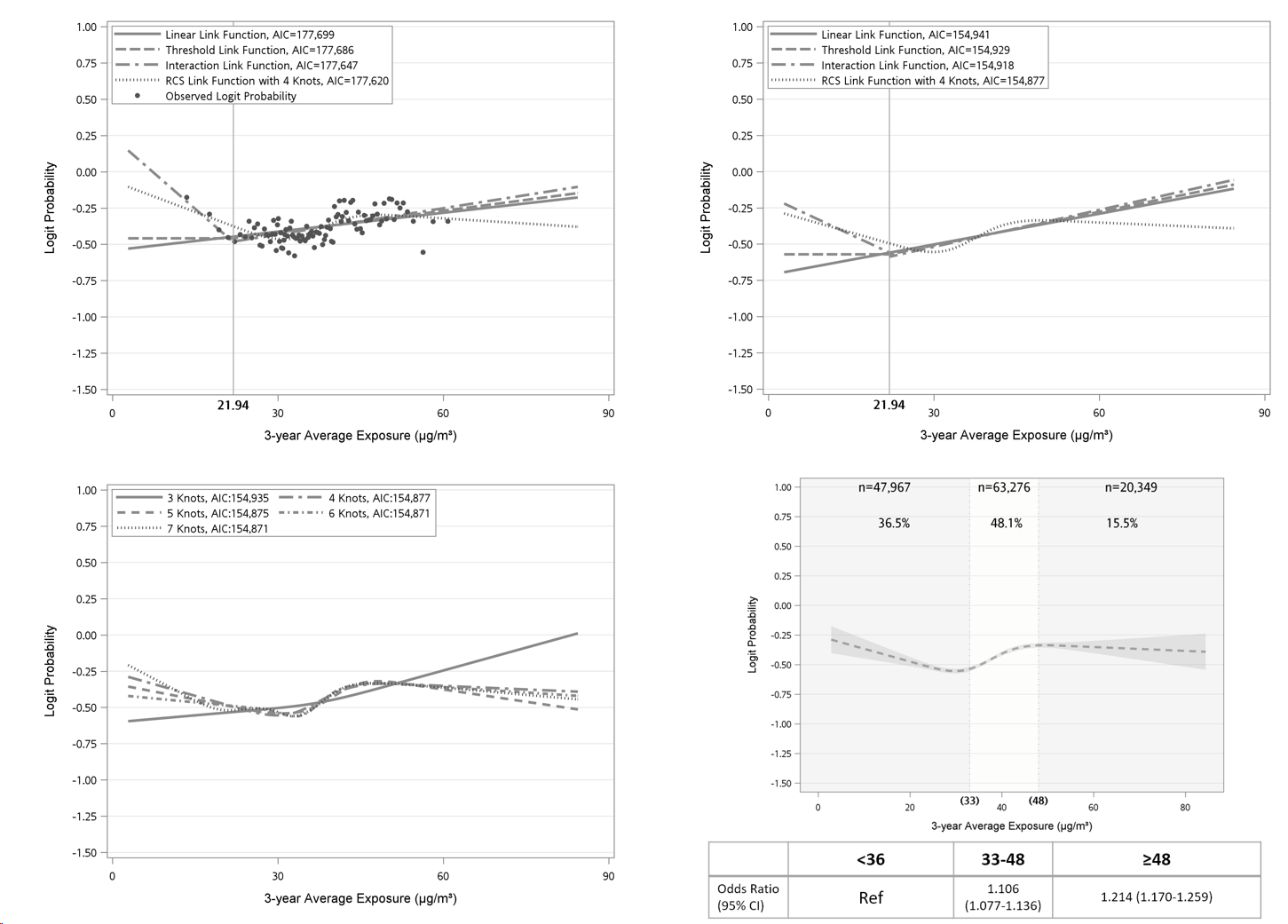


C D

A B

**Fig. S5** Observed and predicted logit probability in association with 3-year average of nitrogen dioxide (NO_2_) exposure for metabolic dysfunction-associated steatotic liver disease (MASLD): (A) Scatter plot of the observed logit probability and comparisons of the predicted logit probability by various crude models: linear (proportional odds) link function; threshold link function; interaction link function; and restricted cubic spline (RCS) link function; (B) Comparisons of predicted logit probability by covariate adjusted models with various link functions; (C) Comparisons of predicted logit probability by covariate adjusted models of RCS link functions of 3 to 7 knots; and (D) The model with “4-knot RCS function” was finally selected to analyze the covariate adjusted logit probability in association with 3-year average exposure of NO_2_ ; and categorization of exposure was determined using cut-off values based on the curve of the graph in the calculation of the covariate adjusted odds ratio and 95% confidence interval (CI) of MASLD. The models in **Fig. S5B, S5C** and **S5D** were adjusted for age, sex, marriage, education, household income, alcohol, smoking, fried food intake, vegetable intake, fruit intake, sugary drink intake, habit of regular exercise and the year of participant enrollment.


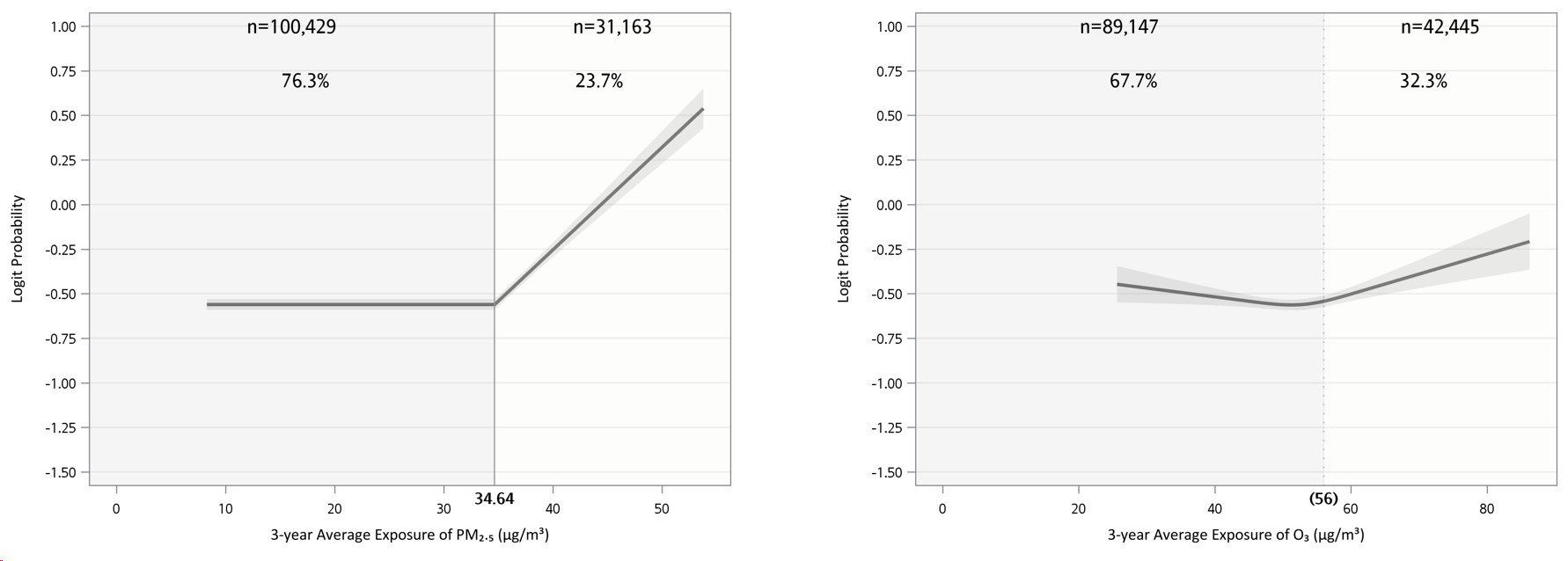


C D

A B


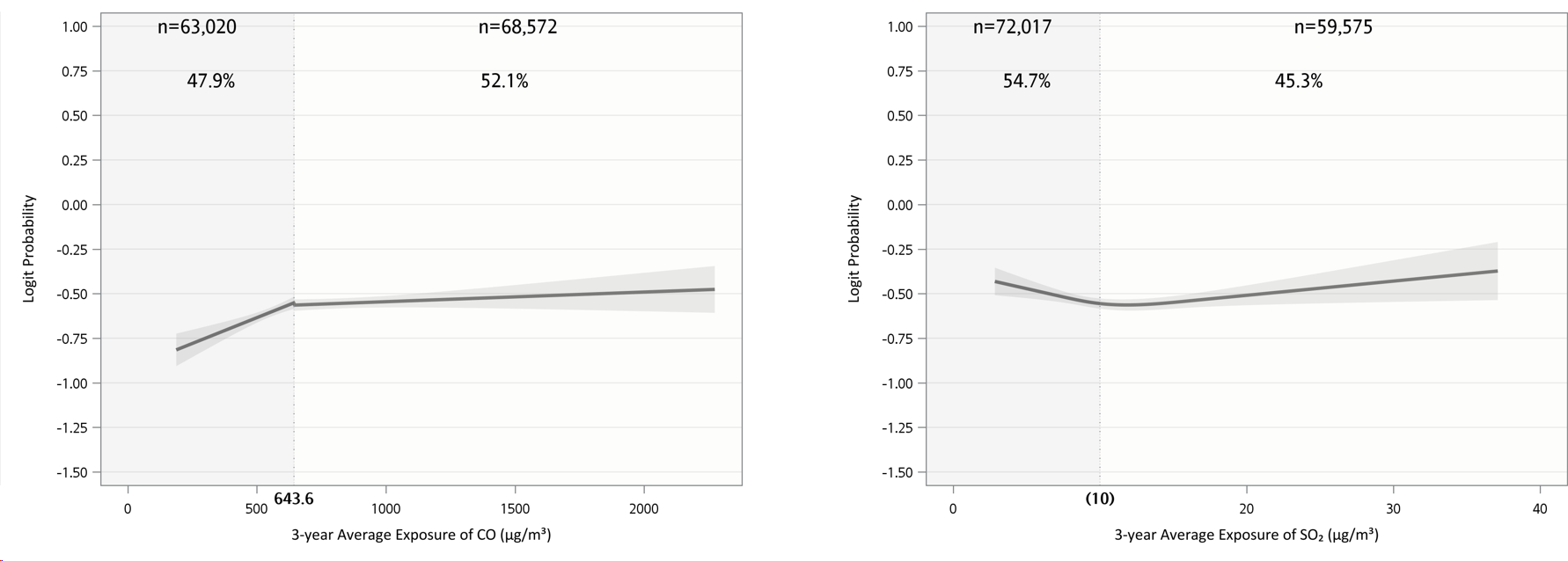


E


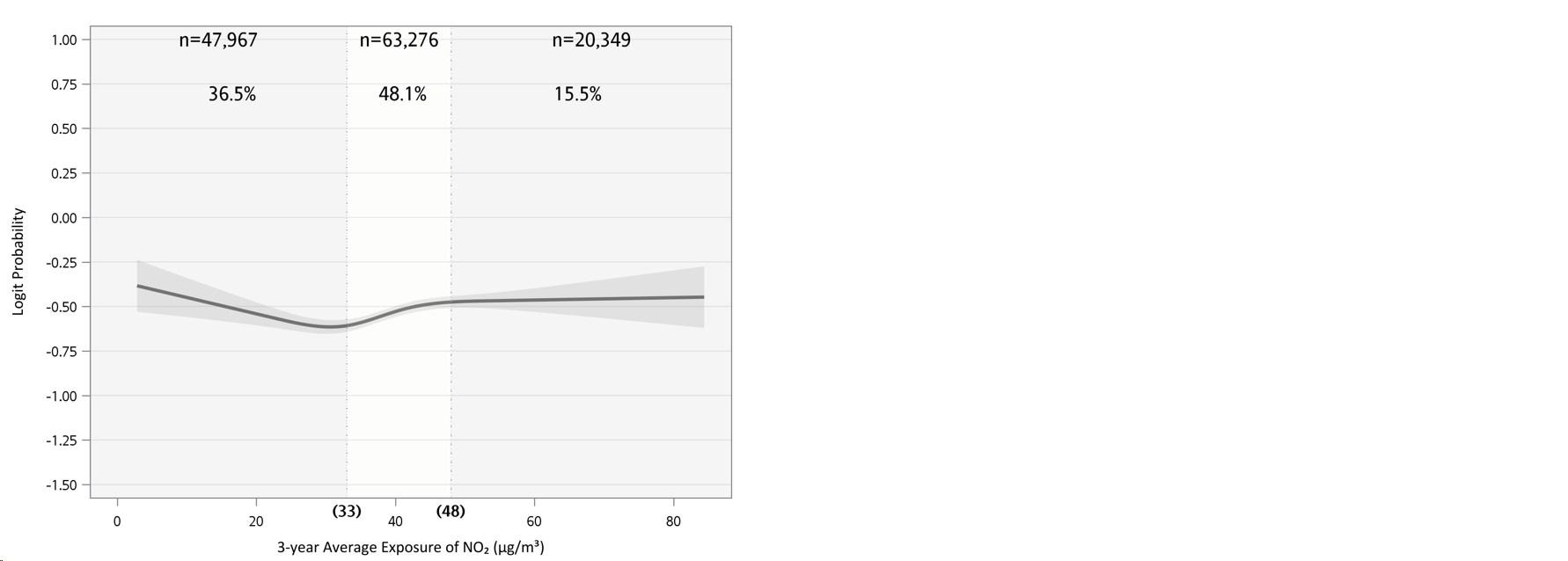


**Fig. S6** Logit probability predicted from covariates adjusted five-pollutant model in association with 3-year average exposure to 5 air pollutants for metabolic dysfunction-associated steatotic liver disease (MASLD): (A) the “threshold link function” for particulate matter with an aerodynamic diameter ≤ 2.5μm, PM_2.5_; (B) the “restricted cubic spline (RCS) link function” with 3 knots for ozone, O_3_; (C) the “interaction link function” for carbon monoxide, CO; (D) the “RCS link function” with 3 knots for sulfur dioxide, SO_2_; and (E) the “RCS” with 4 knots for nitrogen dioxide, NO_2_. The model was adjusted for age, sex, marriage, education, household income, alcohol, smoking, fried food intake, vegetable intake, fruit intake, sugary drink intake, habit of regular exercise and the year of participant enrollment. The plots were done by adjust covariates and other pollutants to the mean value. The cut-off value in **Fig. S6A** and **S6C** were the inflection value based on nonlinear regression, while cut-off values in **Fig.** **S6B, S6D** and **S6E** were based on observation of restricted cubic spline plots.
